# Corkscrew angiopathy of intracranial vessels in a young stroke patient: a case report

**DOI:** 10.1186/1752-1947-6-358

**Published:** 2012-10-23

**Authors:** Anand Alurkar, Lakshmi Sudha P Karanam, Sagar P Oak

**Affiliations:** 1 Department of Neurointervention, KEM Hospital, Pune, India

**Keywords:** Corkscrew angiopathy, Stroke, Digital subtraction angiography

## Abstract

**Introduction:**

We present a rare finding of a ‘corkscrew appearance’ of the distal cerebral vessels in a young Asian woman who presented with acute stroke.

**Case presentation:**

A 32-year-old Asian woman presented with a 3-month history of recurrent right-sided transient ischemic attacks. Her clinical workup and brain imaging results were normal. A digital subtraction angiogram revealed an abnormal corkscrew appearance of all intracranial distal vessels. She was discharged on a single antiplatelet drug. She had no further transient ischemic attacks on clinical follow-up. A digital subtraction angiogram performed 1 year later revealed no changes in the appearance of these vessels.

**Conclusion:**

To the best of our knowledge no similar previous reports exist in the literature. The present report describes a unique case of an unusual corkscrew appearance of the distal intracranial vessels. However, the underlying etiology in the present case remains unknown.

## Introduction

We present a case of a young Asian woman who presented with recurrent right-sided transient ischemic attacks (TIAs) and no neurological deficits. A full screening workup was performed for ‘young stroke’, and all investigation results were within normal limits. Brain imaging revealed no abnormal findings. A digital subtraction angiogram (DSA) showed a diffuse corkscrew appearance of all distal vessels of the cerebral vasculature. We found no clinical associations for the same appearance. To the best of our knowledge this is the first reported case with this appearance of the cerebral vessels.

## Case presentation

**Figure 1:**
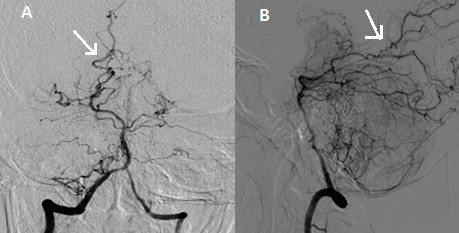
**Vertebral angiogram.** Vertebral angiogram showing the abnormal pattern of the distal vessels of the posterior circulation in both (**A**) anteroposterior and (**B**) lateral projections.

**Figure 2:**
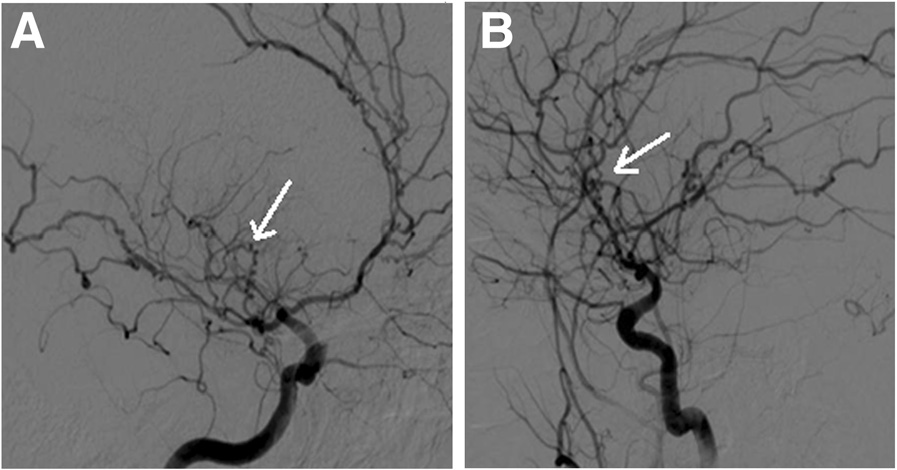
**Right internal carotid artery angiogram.** Digital subtraction angiogram showing the corkscrew appearance of the intracranial small vessels of the right internal carotid angiogram in (**A**) anteroposterior and (**B**) lateral projections.

**Figure 3:**
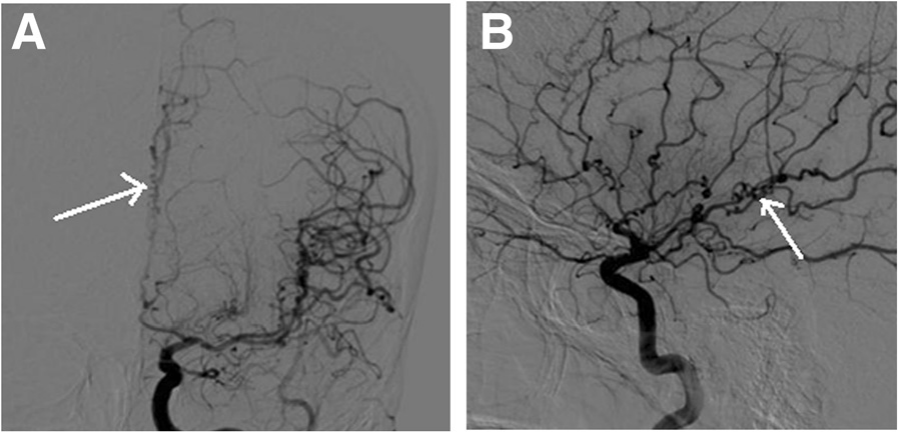
**Left internal carotid artery angiogram.** Digital subtraction angiogram showing corkscrew appearance of the distal small vessels of the left internal carotid angiogram in (**A**) anteroposterior and (**B**) lateral projections.

A 32-year-old right-handed Asian woman presented with recurrent 15- to 20-minute episodes of right upper limb weakness and paresthesia (recurrent TIAs) over a period of 3 months. On clinical examination, all her peripheral pulses were normal, and there were no focal neurological deficits. The patient underwent full urine and blood screening for vasculitis, and the results were negative or within normal limits. Two-dimensional echocardiography results were normal, and no other associated clinical conditions were present. No significant family history was noted. There was no history of any drug intake or exposure to toxins prior to the onset of the symptoms. The results of magnetic resonance imaging (MRI) of the brain were normal. Cerebral DSA showed an abnormal corkscrew appearance of all intracranial distal vessels (Figures 1, 2 and 3) in both the anterior and posterior circulation. There was no evidence of aneurysm or any other vascular malformation. The venous sinuses were normal. Retinal photography was normal. The patient was discharged on a single antiplatelet drug and advised to return for clinical follow-up. At the 1- and 3-month clinical follow-ups, the patient had no history of further episodes of TIAs. A 1-year follow-up angiogram revealed no changes in the corkscrew appearance of the vessels compared to the previous angiogram. The patient had experienced no additional TIAs at the time of the 2-year follow-up.

## Discussion

In a review of the literature, we found no previous reports of an abnormal corkscrew appearance involving only the intracranial small vessels presenting with stroke in young patients. Osborn and Anderson [1] reported a ‘string of beads’ appearance of the vessels involving the intracranial internal carotid artery and middle cerebral artery in their series of 25 patients with fibromuscular dysplasia (FMD). Four patients in their series had FMD involving all four intracranial vessels. However, the present patient showed no evidence of FMD. The appearance of the angiogram suggested an abnormality of the small vessels. Results of a workup for associated vasculitis were negative, and superficial temporal artery biopsy results were normal. Puca *et al*. [2] reported a case of a 32-year-old woman with right brachiocrural hemiparesis who had suffered from left hemispheric ischemic stroke with intracranial arterial dolichoectasia. On a three-dimensional angiogram they demonstrated dolichoectasia of the middle cerebral artery with an unusual corkscrew appearance. Isolated intracranial FMD presenting as a stroke in a 19-year-old woman was reported by Birnbaum *et al*. [3], and a cerebral angiogram showed a string of beads abnormality of the proximal middle cerebral artery and anterior cerebral artery, consistent with FMD. A corkscrew appearance of the retinal vessels was reported in 12 of 32 patients (n=32) with neurofibromatosis type 1 [4]. To the best of our knowledge a similar appearance of the small intracranial vessels associated with other conditions has not been reported in the literature.

Dissection as a cause of the present radiological findings was considered because it is an often underdiagnosed cause of stroke in young patients. However, diffuse involvement with a corkscrew appearance of almost all small intracranial vessels makes dissection a very unlikely etiology. Moreover, the typical angiographic findings of intracranial vessel dissection, such as alternating stenosis and dilatation, delayed arterial emptying, and anastomoses, a beaded appearance, or pseudoaneurysm [5], were not seen in any vessels in our case. The presence of normal MRI results usually rules out underlying vasculitis [6].

It is likely that the DSA findings were incidental and unrelated to the TIAs in the present case. However, in the absence of any other possible cause of the TIAs, a causative association cannot be ruled out. Coutts *et al*. [7] recently reported a case of intracerebral hemorrhage in a young man who presented with stroke. A cerebral angiogram was performed to rule out rare conditions and showed an abnormal corkscrew appearance of the distal vessels that was not typical of any condition. A literature review by these authors suggested the possibility of COL4A1 mutation, and further genetic consultation was done with deoxyribonucleic acid (DNA) sequencing of a COL4A1 complementary DNA product obtained by reverse transcriptase polymerase chain reaction. This investigation evidentially showed a mutation-associated lack of the exon 25 sequence between exons 24 and 26. However, such genetic studies were not performed in the present case because of difficult access to clinical genetic testing centers.

## Conclusion

We report a possibly unique case of an unusual corkscrew appearance of the small vessels of the intracranial circulation in a young woman who presented with stroke and without any underlying known clinical conditions or associations.

## Consent

Written informed consent was obtained from the patient for publication of this case report and accompanying images. A copy of the written consent is available for review by the Editor-in-Chief of this journal.

## Competing interests

The authors declare that they have no competing interests.

## Authors' contributions

AA analyzed and interpreted the patient data. LSPK also analyzed the data and performed the literature review on these findings and contributed to preparation of the manuscript. SPO assisted in the clinical workup and follow-up of the patient as well as the editing and review of the manuscript. All authors read and approved the final manuscript.
